# Supplementary figures and images for: Choice of pre-processing pipeline influences clustering quality of scRNA-seq datasets
Source: BMC Genomics. 2021 Sep 14;22:661. doi: 10.1186/s12864-021-07930-6 (PMC8439043; doi:10.1186/s12864-021-07930-6)

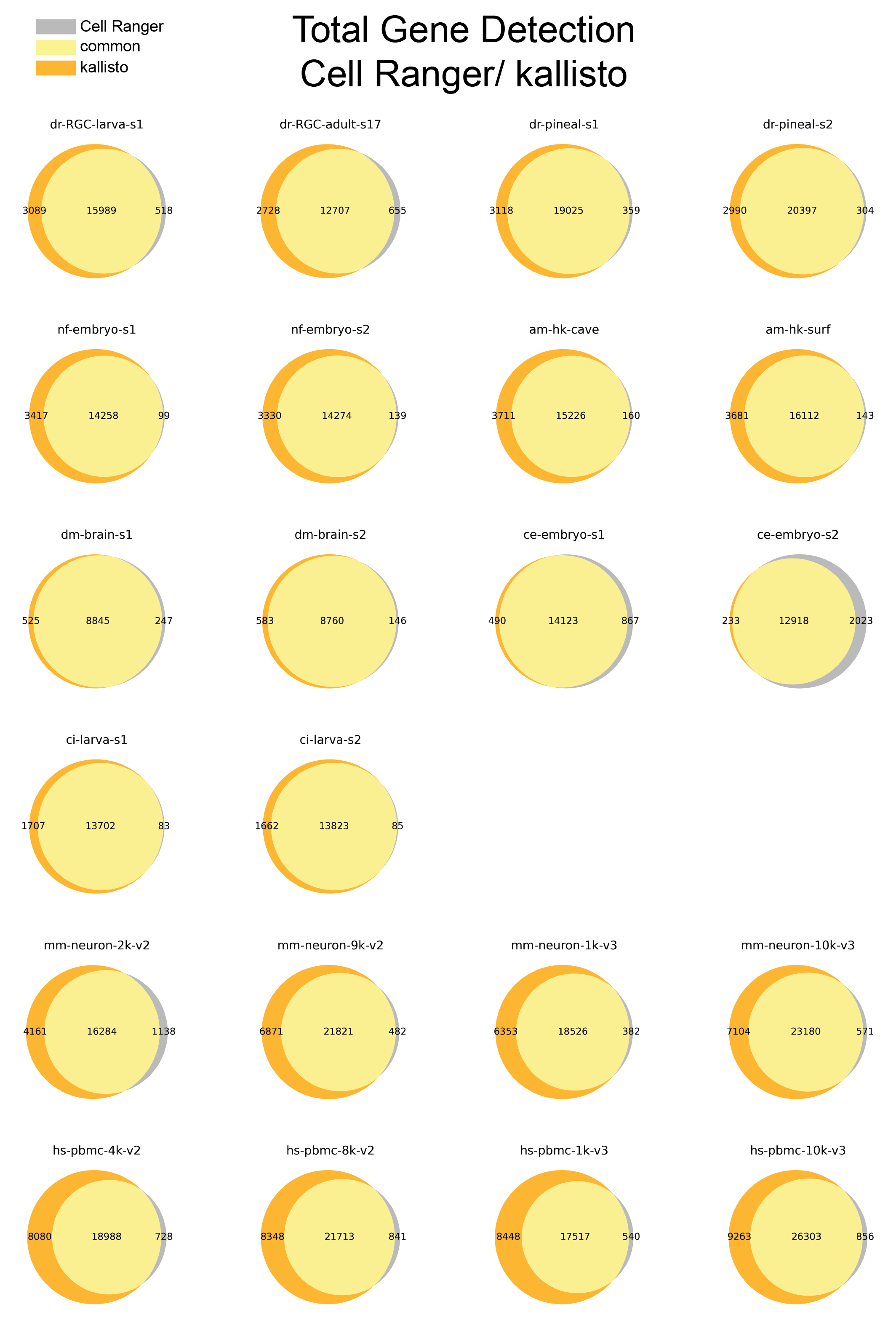

Supplement: Supplementary file 1 — Additional file 1: Fig. S1 Total gene detection of all datasets compared after processing with either kallisto or Cell Ranger. The Venn diagrams show commonly detected number of genes by both pipelines and uniquely detected genes. Fig. S2 Violin-plots showing distribution of gene and UMI detection per cell of all the analyzed datasets (Table 1) run with the Cell Ranger pipeline. Fig. S3 Violin-plots showing distribution of gene and UMI detection per cell of all the analyzed datasets (Table 1) run with the kallisto pipeline. Fig. S4 Cell counts of all datasets compared after processing with either kallisto forced or Cell Ranger. The Venn diagrams show commonly detected cell barcodes by both pipelines and uniquely detected cell barcodes. Fig. S5 Alignment results of all datasets (Table 1) run with either Cell Ranger or kallisto forced against Ensembl reference. a Percent alignment rates of all reads against the reference transcriptome. b Total gene detection. c Median gene counts over all cells per dataset. d Median UMI counts over all cells per dataset. e Total cell counts of each dataset. Fig. S6 Total gene detection of all datasets compared after processing with either kallisto forced or Cell Ranger. The Venn diagrams show commonly detected number of genes by both pipelines and uniquely detected genes. Fig. S7 Violin-plots showing distribution of gene and UMI detection per cell of all the analyzed datasets (Table 1) run with the kallisto forced pipeline. Fig. S8 Violin-plots showing distribution of gene and UMI detection per cell of the dr_pineal_s2 dataset after additional filtering for downstream analysis. Run with either Cell Ranger (a), kallisto (b) or kallisto forced (c). Fig. S9 Downstream analysis of dr_pineal_s2 before cluster merging. a 2D visualization using UMAP of Cell Ranger analyzed clusters before merging, with resolution equal to 0.9. Each point represents a single cell, colored according to cell type. The cells were clustered into 21 types. b Expre [file 12864_2021_7930_MOESM1_ESM.zip › Figure_S1.png]

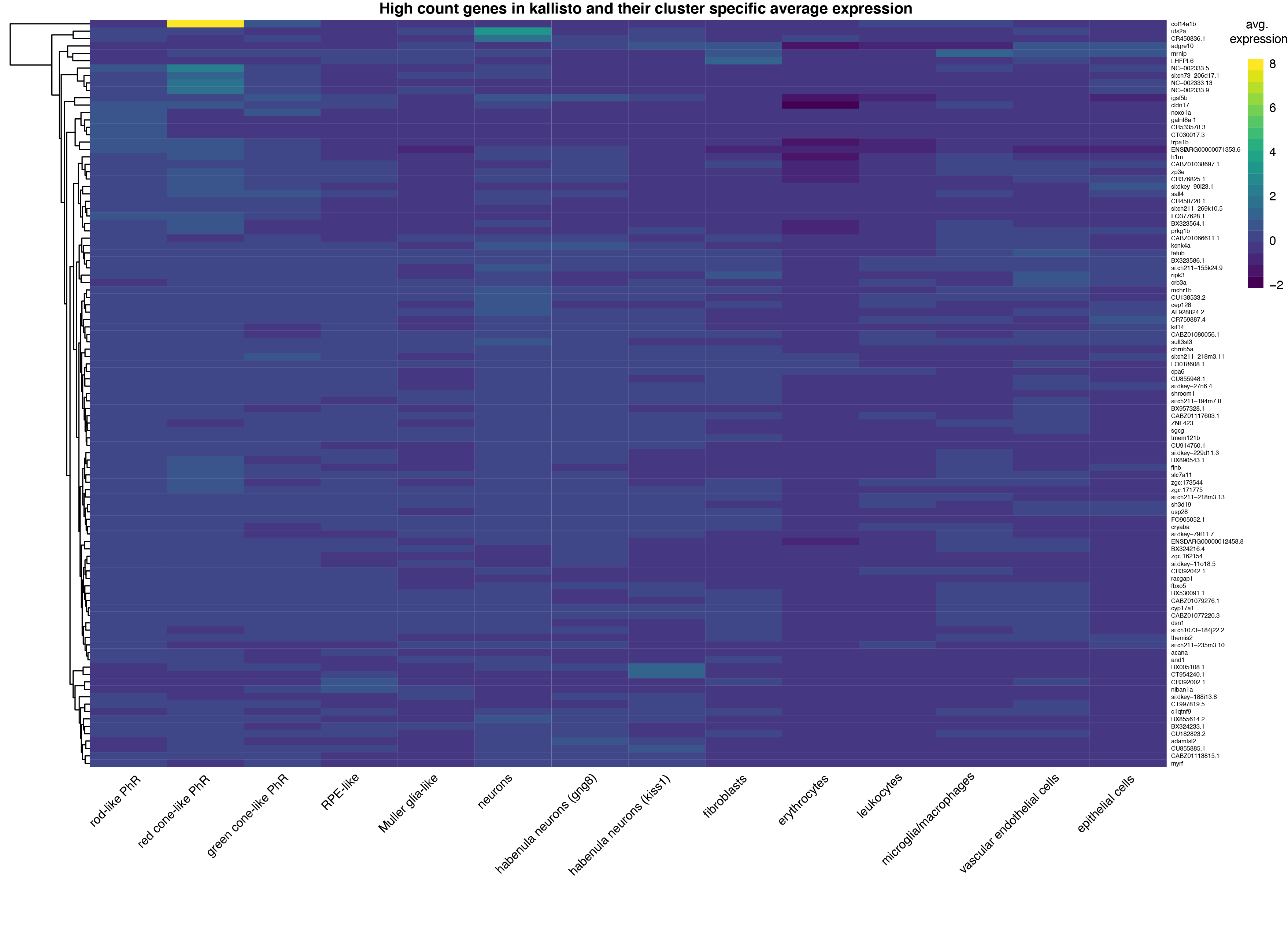

Supplement: Supplementary file 1 — Additional file 1: Fig. S1 Total gene detection of all datasets compared after processing with either kallisto or Cell Ranger. The Venn diagrams show commonly detected number of genes by both pipelines and uniquely detected genes. Fig. S2 Violin-plots showing distribution of gene and UMI detection per cell of all the analyzed datasets (Table 1) run with the Cell Ranger pipeline. Fig. S3 Violin-plots showing distribution of gene and UMI detection per cell of all the analyzed datasets (Table 1) run with the kallisto pipeline. Fig. S4 Cell counts of all datasets compared after processing with either kallisto forced or Cell Ranger. The Venn diagrams show commonly detected cell barcodes by both pipelines and uniquely detected cell barcodes. Fig. S5 Alignment results of all datasets (Table 1) run with either Cell Ranger or kallisto forced against Ensembl reference. a Percent alignment rates of all reads against the reference transcriptome. b Total gene detection. c Median gene counts over all cells per dataset. d Median UMI counts over all cells per dataset. e Total cell counts of each dataset. Fig. S6 Total gene detection of all datasets compared after processing with either kallisto forced or Cell Ranger. The Venn diagrams show commonly detected number of genes by both pipelines and uniquely detected genes. Fig. S7 Violin-plots showing distribution of gene and UMI detection per cell of all the analyzed datasets (Table 1) run with the kallisto forced pipeline. Fig. S8 Violin-plots showing distribution of gene and UMI detection per cell of the dr_pineal_s2 dataset after additional filtering for downstream analysis. Run with either Cell Ranger (a), kallisto (b) or kallisto forced (c). Fig. S9 Downstream analysis of dr_pineal_s2 before cluster merging. a 2D visualization using UMAP of Cell Ranger analyzed clusters before merging, with resolution equal to 0.9. Each point represents a single cell, colored according to cell type. The cells were clustered into 21 types. b Expre [file 12864_2021_7930_MOESM1_ESM.zip › Figure_S10.png]

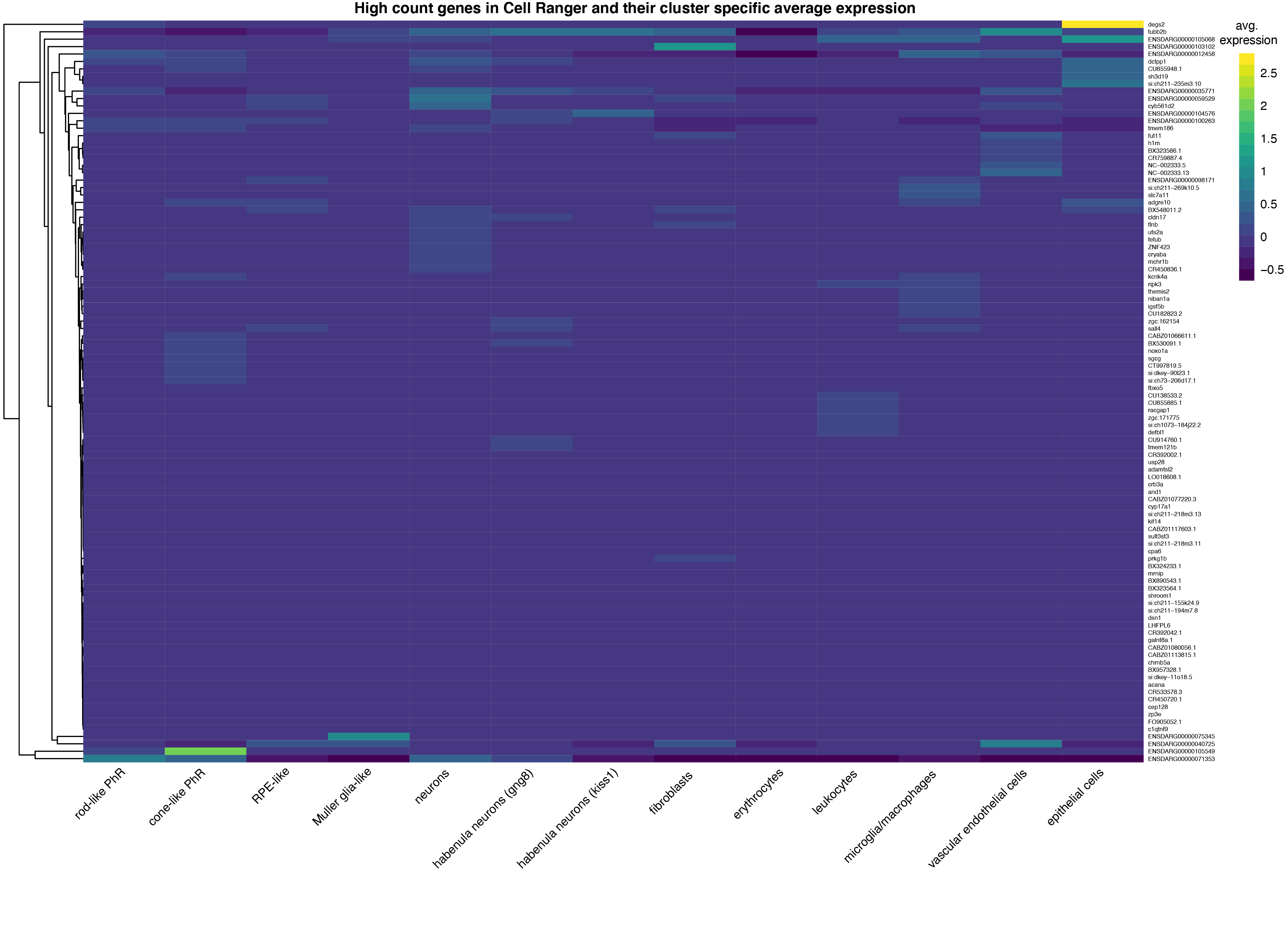

Supplement: Supplementary file 1 — Additional file 1: Fig. S1 Total gene detection of all datasets compared after processing with either kallisto or Cell Ranger. The Venn diagrams show commonly detected number of genes by both pipelines and uniquely detected genes. Fig. S2 Violin-plots showing distribution of gene and UMI detection per cell of all the analyzed datasets (Table 1) run with the Cell Ranger pipeline. Fig. S3 Violin-plots showing distribution of gene and UMI detection per cell of all the analyzed datasets (Table 1) run with the kallisto pipeline. Fig. S4 Cell counts of all datasets compared after processing with either kallisto forced or Cell Ranger. The Venn diagrams show commonly detected cell barcodes by both pipelines and uniquely detected cell barcodes. Fig. S5 Alignment results of all datasets (Table 1) run with either Cell Ranger or kallisto forced against Ensembl reference. a Percent alignment rates of all reads against the reference transcriptome. b Total gene detection. c Median gene counts over all cells per dataset. d Median UMI counts over all cells per dataset. e Total cell counts of each dataset. Fig. S6 Total gene detection of all datasets compared after processing with either kallisto forced or Cell Ranger. The Venn diagrams show commonly detected number of genes by both pipelines and uniquely detected genes. Fig. S7 Violin-plots showing distribution of gene and UMI detection per cell of all the analyzed datasets (Table 1) run with the kallisto forced pipeline. Fig. S8 Violin-plots showing distribution of gene and UMI detection per cell of the dr_pineal_s2 dataset after additional filtering for downstream analysis. Run with either Cell Ranger (a), kallisto (b) or kallisto forced (c). Fig. S9 Downstream analysis of dr_pineal_s2 before cluster merging. a 2D visualization using UMAP of Cell Ranger analyzed clusters before merging, with resolution equal to 0.9. Each point represents a single cell, colored according to cell type. The cells were clustered into 21 types. b Expre [file 12864_2021_7930_MOESM1_ESM.zip › Figure_S11.png]

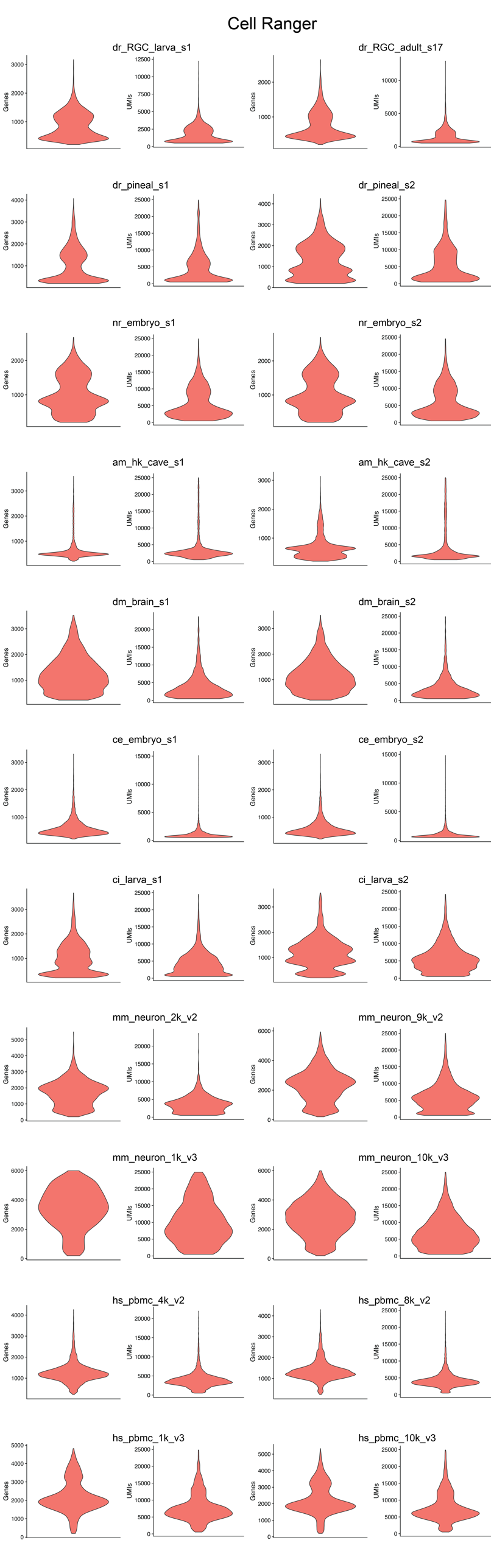

Supplement: Supplementary file 1 — Additional file 1: Fig. S1 Total gene detection of all datasets compared after processing with either kallisto or Cell Ranger. The Venn diagrams show commonly detected number of genes by both pipelines and uniquely detected genes. Fig. S2 Violin-plots showing distribution of gene and UMI detection per cell of all the analyzed datasets (Table 1) run with the Cell Ranger pipeline. Fig. S3 Violin-plots showing distribution of gene and UMI detection per cell of all the analyzed datasets (Table 1) run with the kallisto pipeline. Fig. S4 Cell counts of all datasets compared after processing with either kallisto forced or Cell Ranger. The Venn diagrams show commonly detected cell barcodes by both pipelines and uniquely detected cell barcodes. Fig. S5 Alignment results of all datasets (Table 1) run with either Cell Ranger or kallisto forced against Ensembl reference. a Percent alignment rates of all reads against the reference transcriptome. b Total gene detection. c Median gene counts over all cells per dataset. d Median UMI counts over all cells per dataset. e Total cell counts of each dataset. Fig. S6 Total gene detection of all datasets compared after processing with either kallisto forced or Cell Ranger. The Venn diagrams show commonly detected number of genes by both pipelines and uniquely detected genes. Fig. S7 Violin-plots showing distribution of gene and UMI detection per cell of all the analyzed datasets (Table 1) run with the kallisto forced pipeline. Fig. S8 Violin-plots showing distribution of gene and UMI detection per cell of the dr_pineal_s2 dataset after additional filtering for downstream analysis. Run with either Cell Ranger (a), kallisto (b) or kallisto forced (c). Fig. S9 Downstream analysis of dr_pineal_s2 before cluster merging. a 2D visualization using UMAP of Cell Ranger analyzed clusters before merging, with resolution equal to 0.9. Each point represents a single cell, colored according to cell type. The cells were clustered into 21 types. b Expre [file 12864_2021_7930_MOESM1_ESM.zip › Figure_S2.png]

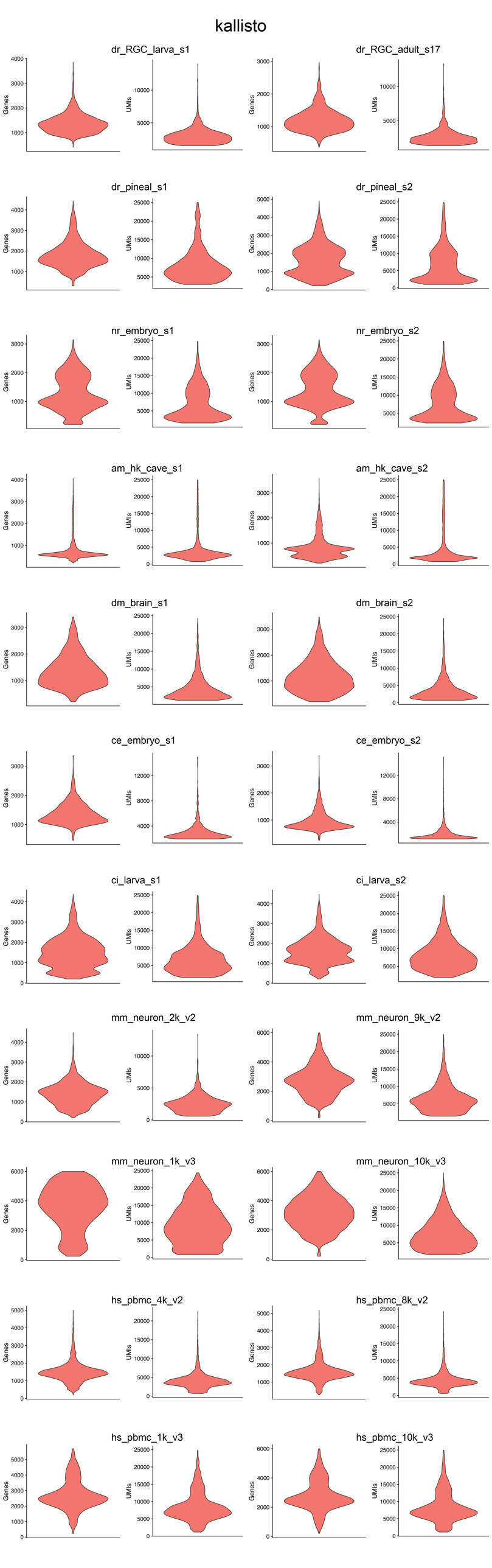

Supplement: Supplementary file 1 — Additional file 1: Fig. S1 Total gene detection of all datasets compared after processing with either kallisto or Cell Ranger. The Venn diagrams show commonly detected number of genes by both pipelines and uniquely detected genes. Fig. S2 Violin-plots showing distribution of gene and UMI detection per cell of all the analyzed datasets (Table 1) run with the Cell Ranger pipeline. Fig. S3 Violin-plots showing distribution of gene and UMI detection per cell of all the analyzed datasets (Table 1) run with the kallisto pipeline. Fig. S4 Cell counts of all datasets compared after processing with either kallisto forced or Cell Ranger. The Venn diagrams show commonly detected cell barcodes by both pipelines and uniquely detected cell barcodes. Fig. S5 Alignment results of all datasets (Table 1) run with either Cell Ranger or kallisto forced against Ensembl reference. a Percent alignment rates of all reads against the reference transcriptome. b Total gene detection. c Median gene counts over all cells per dataset. d Median UMI counts over all cells per dataset. e Total cell counts of each dataset. Fig. S6 Total gene detection of all datasets compared after processing with either kallisto forced or Cell Ranger. The Venn diagrams show commonly detected number of genes by both pipelines and uniquely detected genes. Fig. S7 Violin-plots showing distribution of gene and UMI detection per cell of all the analyzed datasets (Table 1) run with the kallisto forced pipeline. Fig. S8 Violin-plots showing distribution of gene and UMI detection per cell of the dr_pineal_s2 dataset after additional filtering for downstream analysis. Run with either Cell Ranger (a), kallisto (b) or kallisto forced (c). Fig. S9 Downstream analysis of dr_pineal_s2 before cluster merging. a 2D visualization using UMAP of Cell Ranger analyzed clusters before merging, with resolution equal to 0.9. Each point represents a single cell, colored according to cell type. The cells were clustered into 21 types. b Expre [file 12864_2021_7930_MOESM1_ESM.zip › Figure_S3.png]

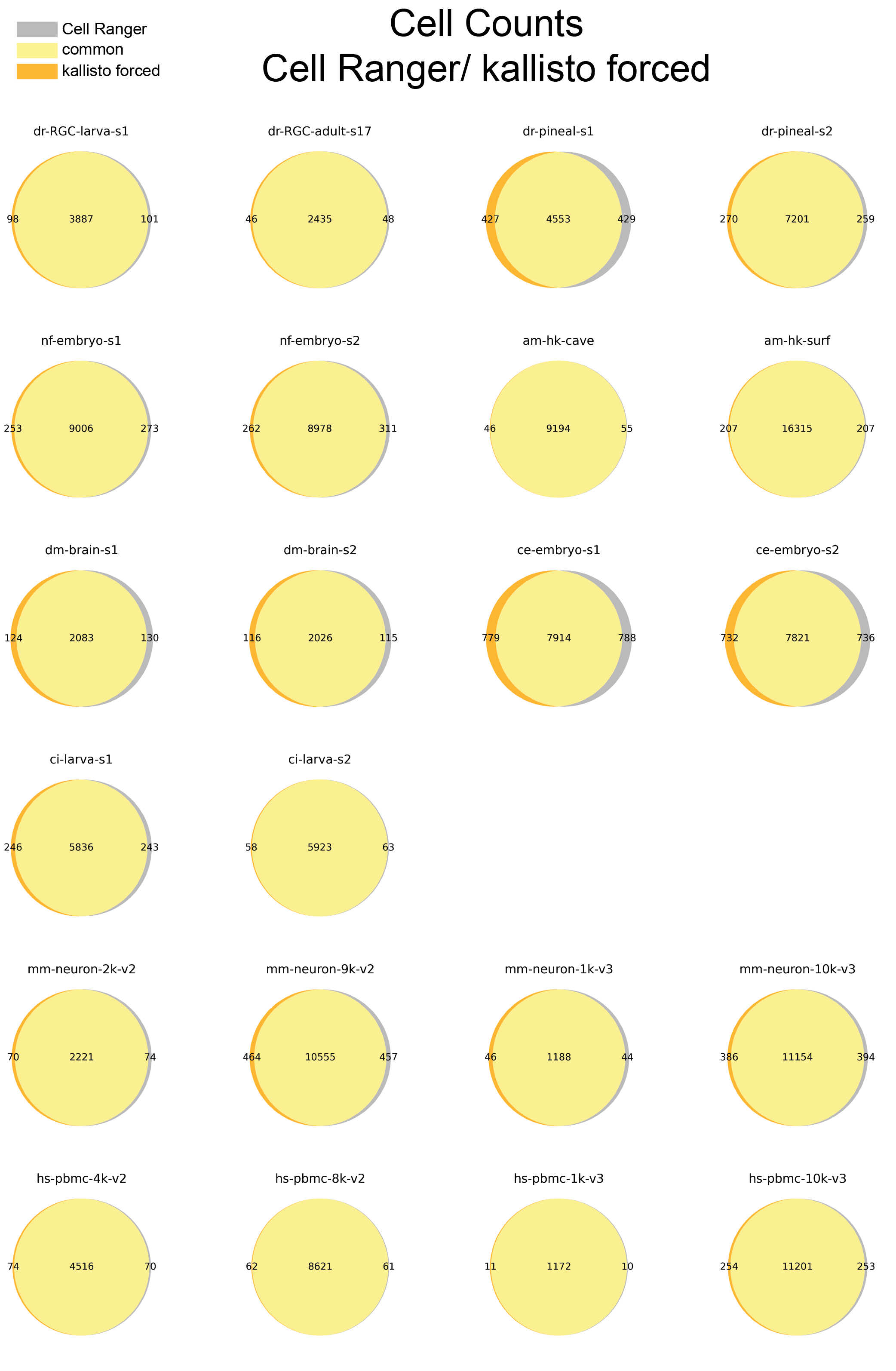

Supplement: Supplementary file 1 — Additional file 1: Fig. S1 Total gene detection of all datasets compared after processing with either kallisto or Cell Ranger. The Venn diagrams show commonly detected number of genes by both pipelines and uniquely detected genes. Fig. S2 Violin-plots showing distribution of gene and UMI detection per cell of all the analyzed datasets (Table 1) run with the Cell Ranger pipeline. Fig. S3 Violin-plots showing distribution of gene and UMI detection per cell of all the analyzed datasets (Table 1) run with the kallisto pipeline. Fig. S4 Cell counts of all datasets compared after processing with either kallisto forced or Cell Ranger. The Venn diagrams show commonly detected cell barcodes by both pipelines and uniquely detected cell barcodes. Fig. S5 Alignment results of all datasets (Table 1) run with either Cell Ranger or kallisto forced against Ensembl reference. a Percent alignment rates of all reads against the reference transcriptome. b Total gene detection. c Median gene counts over all cells per dataset. d Median UMI counts over all cells per dataset. e Total cell counts of each dataset. Fig. S6 Total gene detection of all datasets compared after processing with either kallisto forced or Cell Ranger. The Venn diagrams show commonly detected number of genes by both pipelines and uniquely detected genes. Fig. S7 Violin-plots showing distribution of gene and UMI detection per cell of all the analyzed datasets (Table 1) run with the kallisto forced pipeline. Fig. S8 Violin-plots showing distribution of gene and UMI detection per cell of the dr_pineal_s2 dataset after additional filtering for downstream analysis. Run with either Cell Ranger (a), kallisto (b) or kallisto forced (c). Fig. S9 Downstream analysis of dr_pineal_s2 before cluster merging. a 2D visualization using UMAP of Cell Ranger analyzed clusters before merging, with resolution equal to 0.9. Each point represents a single cell, colored according to cell type. The cells were clustered into 21 types. b Expre [file 12864_2021_7930_MOESM1_ESM.zip › Figure_S4.png]

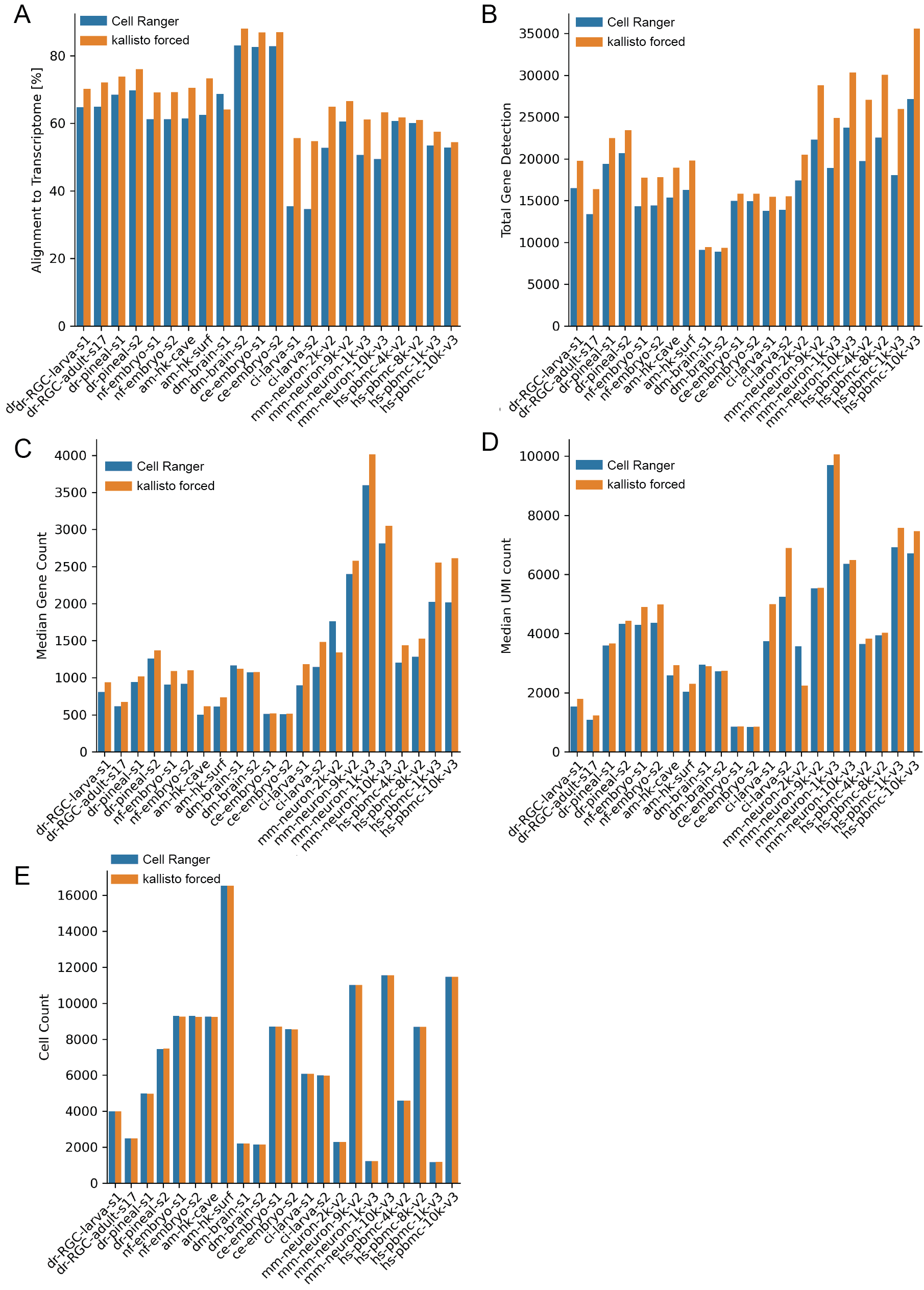

Supplement: Supplementary file 1 — Additional file 1: Fig. S1 Total gene detection of all datasets compared after processing with either kallisto or Cell Ranger. The Venn diagrams show commonly detected number of genes by both pipelines and uniquely detected genes. Fig. S2 Violin-plots showing distribution of gene and UMI detection per cell of all the analyzed datasets (Table 1) run with the Cell Ranger pipeline. Fig. S3 Violin-plots showing distribution of gene and UMI detection per cell of all the analyzed datasets (Table 1) run with the kallisto pipeline. Fig. S4 Cell counts of all datasets compared after processing with either kallisto forced or Cell Ranger. The Venn diagrams show commonly detected cell barcodes by both pipelines and uniquely detected cell barcodes. Fig. S5 Alignment results of all datasets (Table 1) run with either Cell Ranger or kallisto forced against Ensembl reference. a Percent alignment rates of all reads against the reference transcriptome. b Total gene detection. c Median gene counts over all cells per dataset. d Median UMI counts over all cells per dataset. e Total cell counts of each dataset. Fig. S6 Total gene detection of all datasets compared after processing with either kallisto forced or Cell Ranger. The Venn diagrams show commonly detected number of genes by both pipelines and uniquely detected genes. Fig. S7 Violin-plots showing distribution of gene and UMI detection per cell of all the analyzed datasets (Table 1) run with the kallisto forced pipeline. Fig. S8 Violin-plots showing distribution of gene and UMI detection per cell of the dr_pineal_s2 dataset after additional filtering for downstream analysis. Run with either Cell Ranger (a), kallisto (b) or kallisto forced (c). Fig. S9 Downstream analysis of dr_pineal_s2 before cluster merging. a 2D visualization using UMAP of Cell Ranger analyzed clusters before merging, with resolution equal to 0.9. Each point represents a single cell, colored according to cell type. The cells were clustered into 21 types. b Expre [file 12864_2021_7930_MOESM1_ESM.zip › Figure_S5.png]

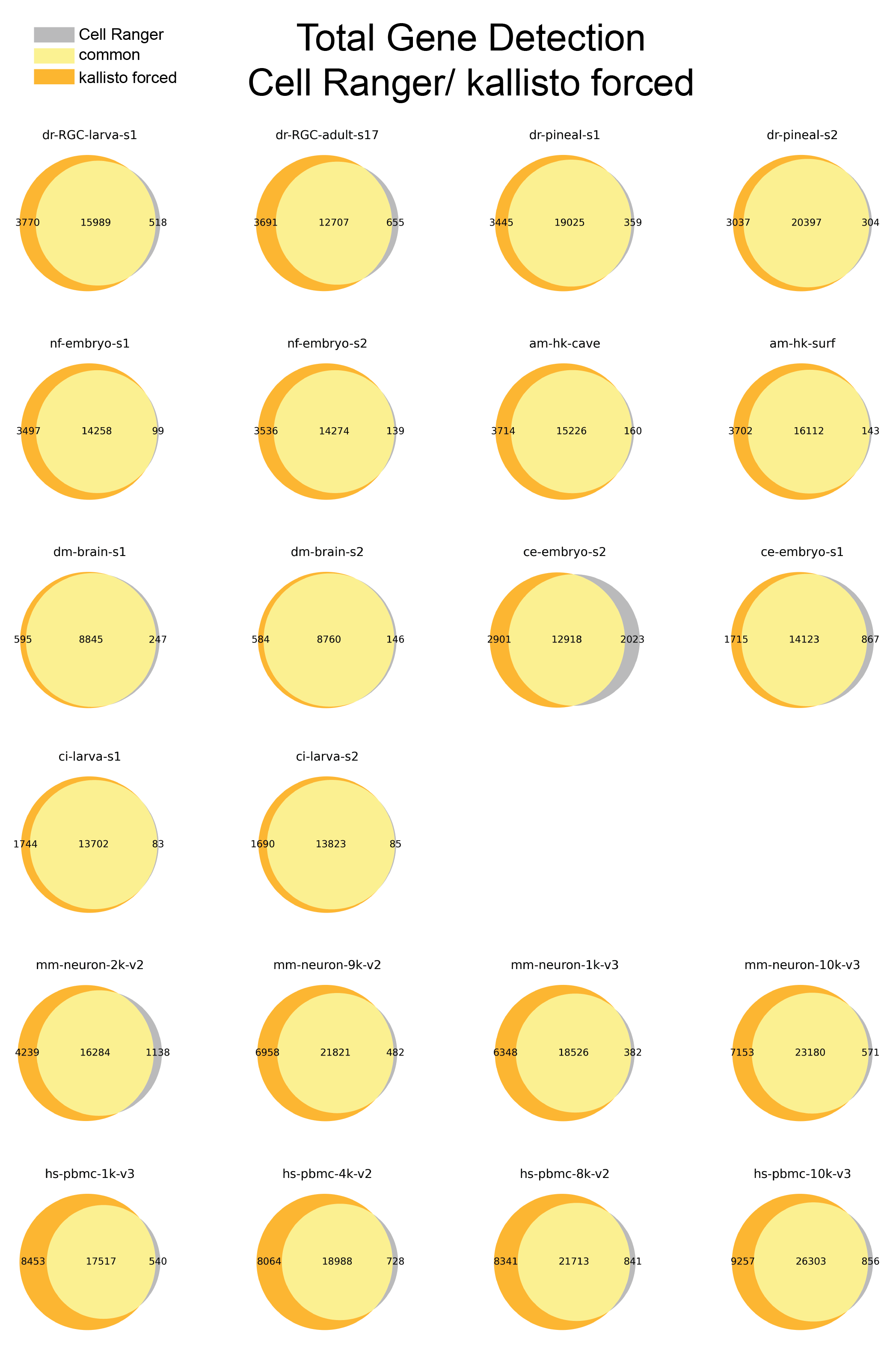

Supplement: Supplementary file 1 — Additional file 1: Fig. S1 Total gene detection of all datasets compared after processing with either kallisto or Cell Ranger. The Venn diagrams show commonly detected number of genes by both pipelines and uniquely detected genes. Fig. S2 Violin-plots showing distribution of gene and UMI detection per cell of all the analyzed datasets (Table 1) run with the Cell Ranger pipeline. Fig. S3 Violin-plots showing distribution of gene and UMI detection per cell of all the analyzed datasets (Table 1) run with the kallisto pipeline. Fig. S4 Cell counts of all datasets compared after processing with either kallisto forced or Cell Ranger. The Venn diagrams show commonly detected cell barcodes by both pipelines and uniquely detected cell barcodes. Fig. S5 Alignment results of all datasets (Table 1) run with either Cell Ranger or kallisto forced against Ensembl reference. a Percent alignment rates of all reads against the reference transcriptome. b Total gene detection. c Median gene counts over all cells per dataset. d Median UMI counts over all cells per dataset. e Total cell counts of each dataset. Fig. S6 Total gene detection of all datasets compared after processing with either kallisto forced or Cell Ranger. The Venn diagrams show commonly detected number of genes by both pipelines and uniquely detected genes. Fig. S7 Violin-plots showing distribution of gene and UMI detection per cell of all the analyzed datasets (Table 1) run with the kallisto forced pipeline. Fig. S8 Violin-plots showing distribution of gene and UMI detection per cell of the dr_pineal_s2 dataset after additional filtering for downstream analysis. Run with either Cell Ranger (a), kallisto (b) or kallisto forced (c). Fig. S9 Downstream analysis of dr_pineal_s2 before cluster merging. a 2D visualization using UMAP of Cell Ranger analyzed clusters before merging, with resolution equal to 0.9. Each point represents a single cell, colored according to cell type. The cells were clustered into 21 types. b Expre [file 12864_2021_7930_MOESM1_ESM.zip › Figure_S6.png]

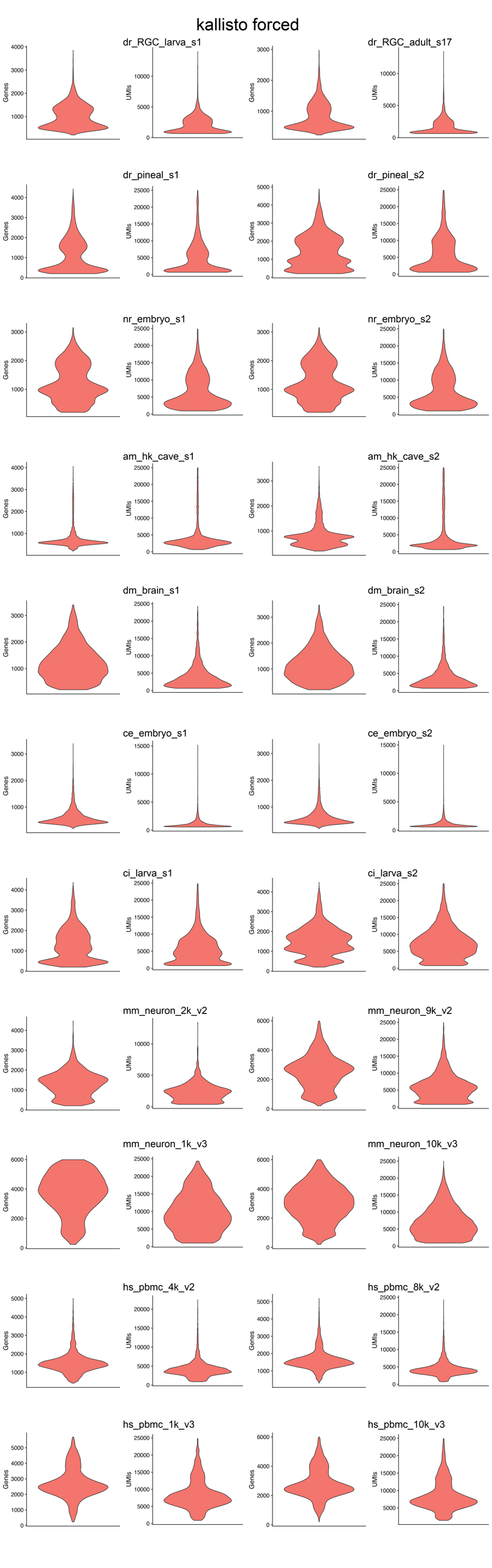

Supplement: Supplementary file 1 — Additional file 1: Fig. S1 Total gene detection of all datasets compared after processing with either kallisto or Cell Ranger. The Venn diagrams show commonly detected number of genes by both pipelines and uniquely detected genes. Fig. S2 Violin-plots showing distribution of gene and UMI detection per cell of all the analyzed datasets (Table 1) run with the Cell Ranger pipeline. Fig. S3 Violin-plots showing distribution of gene and UMI detection per cell of all the analyzed datasets (Table 1) run with the kallisto pipeline. Fig. S4 Cell counts of all datasets compared after processing with either kallisto forced or Cell Ranger. The Venn diagrams show commonly detected cell barcodes by both pipelines and uniquely detected cell barcodes. Fig. S5 Alignment results of all datasets (Table 1) run with either Cell Ranger or kallisto forced against Ensembl reference. a Percent alignment rates of all reads against the reference transcriptome. b Total gene detection. c Median gene counts over all cells per dataset. d Median UMI counts over all cells per dataset. e Total cell counts of each dataset. Fig. S6 Total gene detection of all datasets compared after processing with either kallisto forced or Cell Ranger. The Venn diagrams show commonly detected number of genes by both pipelines and uniquely detected genes. Fig. S7 Violin-plots showing distribution of gene and UMI detection per cell of all the analyzed datasets (Table 1) run with the kallisto forced pipeline. Fig. S8 Violin-plots showing distribution of gene and UMI detection per cell of the dr_pineal_s2 dataset after additional filtering for downstream analysis. Run with either Cell Ranger (a), kallisto (b) or kallisto forced (c). Fig. S9 Downstream analysis of dr_pineal_s2 before cluster merging. a 2D visualization using UMAP of Cell Ranger analyzed clusters before merging, with resolution equal to 0.9. Each point represents a single cell, colored according to cell type. The cells were clustered into 21 types. b Expre [file 12864_2021_7930_MOESM1_ESM.zip › Figure_S7.png]

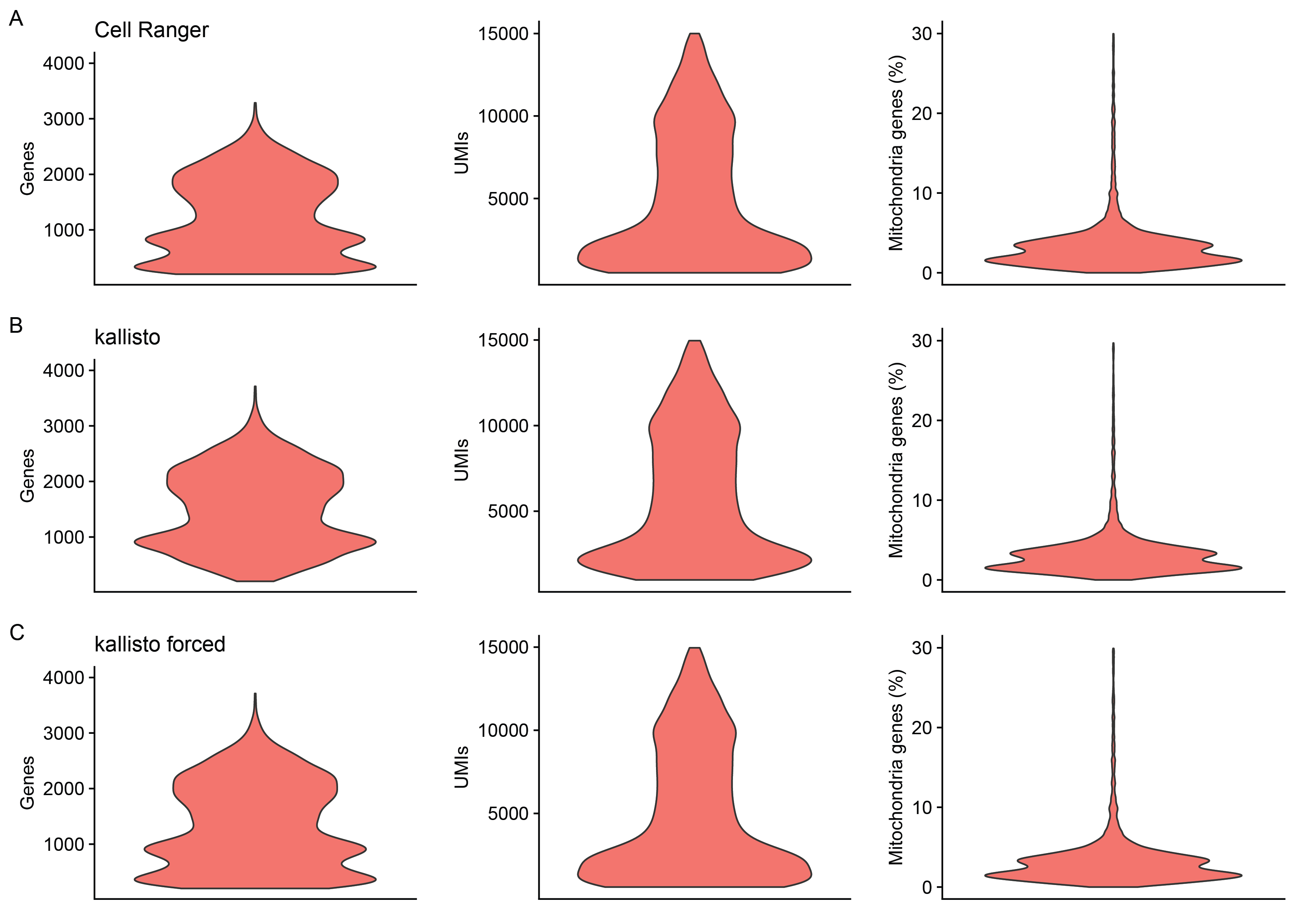

Supplement: Supplementary file 1 — Additional file 1: Fig. S1 Total gene detection of all datasets compared after processing with either kallisto or Cell Ranger. The Venn diagrams show commonly detected number of genes by both pipelines and uniquely detected genes. Fig. S2 Violin-plots showing distribution of gene and UMI detection per cell of all the analyzed datasets (Table 1) run with the Cell Ranger pipeline. Fig. S3 Violin-plots showing distribution of gene and UMI detection per cell of all the analyzed datasets (Table 1) run with the kallisto pipeline. Fig. S4 Cell counts of all datasets compared after processing with either kallisto forced or Cell Ranger. The Venn diagrams show commonly detected cell barcodes by both pipelines and uniquely detected cell barcodes. Fig. S5 Alignment results of all datasets (Table 1) run with either Cell Ranger or kallisto forced against Ensembl reference. a Percent alignment rates of all reads against the reference transcriptome. b Total gene detection. c Median gene counts over all cells per dataset. d Median UMI counts over all cells per dataset. e Total cell counts of each dataset. Fig. S6 Total gene detection of all datasets compared after processing with either kallisto forced or Cell Ranger. The Venn diagrams show commonly detected number of genes by both pipelines and uniquely detected genes. Fig. S7 Violin-plots showing distribution of gene and UMI detection per cell of all the analyzed datasets (Table 1) run with the kallisto forced pipeline. Fig. S8 Violin-plots showing distribution of gene and UMI detection per cell of the dr_pineal_s2 dataset after additional filtering for downstream analysis. Run with either Cell Ranger (a), kallisto (b) or kallisto forced (c). Fig. S9 Downstream analysis of dr_pineal_s2 before cluster merging. a 2D visualization using UMAP of Cell Ranger analyzed clusters before merging, with resolution equal to 0.9. Each point represents a single cell, colored according to cell type. The cells were clustered into 21 types. b Expre [file 12864_2021_7930_MOESM1_ESM.zip › Figure_S8.png]

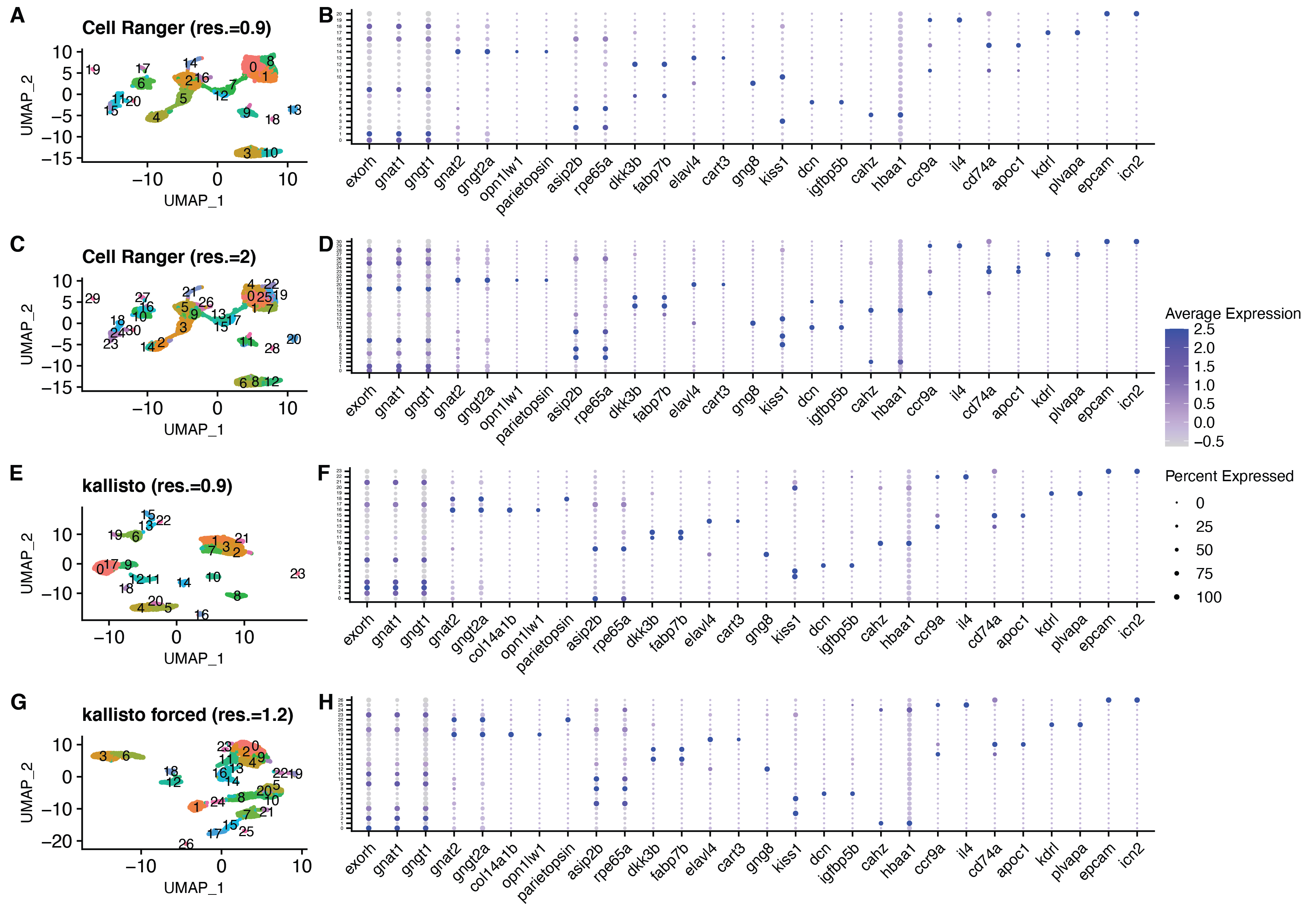

Supplement: Supplementary file 1 — Additional file 1: Fig. S1 Total gene detection of all datasets compared after processing with either kallisto or Cell Ranger. The Venn diagrams show commonly detected number of genes by both pipelines and uniquely detected genes. Fig. S2 Violin-plots showing distribution of gene and UMI detection per cell of all the analyzed datasets (Table 1) run with the Cell Ranger pipeline. Fig. S3 Violin-plots showing distribution of gene and UMI detection per cell of all the analyzed datasets (Table 1) run with the kallisto pipeline. Fig. S4 Cell counts of all datasets compared after processing with either kallisto forced or Cell Ranger. The Venn diagrams show commonly detected cell barcodes by both pipelines and uniquely detected cell barcodes. Fig. S5 Alignment results of all datasets (Table 1) run with either Cell Ranger or kallisto forced against Ensembl reference. a Percent alignment rates of all reads against the reference transcriptome. b Total gene detection. c Median gene counts over all cells per dataset. d Median UMI counts over all cells per dataset. e Total cell counts of each dataset. Fig. S6 Total gene detection of all datasets compared after processing with either kallisto forced or Cell Ranger. The Venn diagrams show commonly detected number of genes by both pipelines and uniquely detected genes. Fig. S7 Violin-plots showing distribution of gene and UMI detection per cell of all the analyzed datasets (Table 1) run with the kallisto forced pipeline. Fig. S8 Violin-plots showing distribution of gene and UMI detection per cell of the dr_pineal_s2 dataset after additional filtering for downstream analysis. Run with either Cell Ranger (a), kallisto (b) or kallisto forced (c). Fig. S9 Downstream analysis of dr_pineal_s2 before cluster merging. a 2D visualization using UMAP of Cell Ranger analyzed clusters before merging, with resolution equal to 0.9. Each point represents a single cell, colored according to cell type. The cells were clustered into 21 types. b Expre [file 12864_2021_7930_MOESM1_ESM.zip › Figure_S9.png]
